# Interactive Guidance Intervention to Address Sustained Social Withdrawal in Preterm Infants in Chile: Protocol for a Randomized Controlled Trial

**DOI:** 10.2196/17943

**Published:** 2020-06-26

**Authors:** Jorge Bustamante Loyola, Marcela Perez Retamal, Monica Isabel Morgues Nudman, Andres Maturana, Ricardo Salinas Gonzalez, Horacio Cox, José Miguel González Mas, Lucia Muñoz, Lilian Lopez, Andrés Mendiburo-Seguel, Sandra Simó, Pascual Palau Subiela, Antoine Guedeney

**Affiliations:** 1 Neonatology Unit Clinica Alemana de Santiago Santiago Chile; 2 Doctoral Programme in Clinical and Health Psychology Universitat de Valencia Valencia Spain; 3 Spain Association for Infant Mental Health Since Gestation Valencia Spain; 4 Neonatology Unit Hospital San Jose Santiago Chile; 5 Faculty Development Office Universidad del Desarrollo Santiago Chile; 6 Faculty of Education and Social Sciences Universidad Andrés Bello Santiago Chile; 7 Faculty of Psychology Universitat de Valencia Valencia Spain; 8 Hospital Bichat Claude Bernard Assistance Publique–Hôpitaux de Paris Paris France; 9 Université Paris Diderot, Paris 7 Paris France

**Keywords:** social withdrawal, preterm, early detection, interactive guidance, emotional stress, social development, postnatal depression, posttraumatic stress

## Abstract

**Background:**

Preterm newborns can be exposed early to significant perinatal stress, and this stress can increase the risk of altered socioemotional development. Sustained social withdrawal in infants is an early indicator of emotional distress which is expressed by low reactivity to the environment, and if persistent, is frequently associated with altered psychological development. Infants born prematurely have a higher probability of developing sustained social withdrawal (adjusted odds ratio 1.84, 95% CI 1.04-3.26) than infants born full term, and there is a correlation between weight at birth and sustained social withdrawal at 12 months of age.

**Objective:**

The aims of this study are to compare the effect of the interactive guidance intervention to that of routine pediatric care on sustained social withdrawal in infants born moderately or late preterm and to explore the relationship between sustained social withdrawal in these infants and factors such as neonatal intensive care unit hospitalization variables, parental depression, and posttraumatic stress symptoms.

**Methods:**

This study is designed as a multicenter randomized controlled trial. Moderate and late preterm newborns and their parents were recruited and randomized (1:1 allocation ratio) to control and experimental groups. During neonatal intensive care unit hospitalization, daily duration of skin-to-skin contact, breastfeeding, and parental visits were recorded. Also, a daily score for neonatal pain and painful invasive procedures were recorded. After discharge from neonatal intensive care, for the duration of the study, both groups will attend follow-up consultations with neonatologists at 2, 6, and 12 months of age (corrected for gestational age) and will receive routine pediatric care. Every consultation will be recorded and assessed with the Alarm Distress Baby Scale to detect sustained social withdrawal (indicated by a score of 5 or higher). The neonatologists will perform an interactive guidance intervention if an infant in the intervention group exhibits sustained social withdrawal. In each follow-up consultation, parents will fill out the Edinburgh Postnatal Depression Scale, the modified Perinatal Posttraumatic Stress Disorder Questionnaire, and the Impact of Event Scale–revised.

**Results:**

Recruitment for this trial started in September 2017. As of May 2020, we have completed enrollment (N=110 infants born moderately or late preterm). We aim to publish the results by mid-2021.

**Conclusions:**

This is the first randomized controlled trial with a sample of infants born moderately or late preterm infants who will attend pediatric follow-up consultations during their first year (corrected for gestational age at birth) with neonatologists trained in the Alarm Distress Baby Scale and who will receive this interactive guidance intervention. If successful, this early intervention will show significant potential to be implemented in both public and private health care, given its low cost of training staff and that the intervention takes place during routine pediatric follow-up.

**Trial Registration:**

ClinicalTrials.gov NCT03212547; https://clinicaltrials.gov/ct2/show/NCT03212547.

**International Registered Report Identifier (IRRID):**

DERR1-10.2196/17943

## Introduction

### Background

In infants in the normal range of development, skills to engage with caregivers such as initiating and maintaining eye contact, vocalizing, using facial expressions, and using body movements, emerge during the first two months after birth [1,2]. The level of synchronization between infants and their caregivers and the capacity of caregivers to detect interactive errors and attune with the infant’s emotional state and interactive behaviors appears to be critical for optimal psychological development in the first 18 months of life [3].

Infants can display social withdrawal behaviors as a reaction to minor transient perturbations when interacting with caregivers or when agitated or tired; however, infants are usually able to reengage as soon as they regain the full attention of the caregivers [4-6]. In contrast with this adaptive social withdrawal behavior, sustained social withdrawal is significantly less common and reflects a sustained decrease in engagement during interactions and a sustained decrease in reactivity to the environment [7,8]. Sustained social withdrawal can be assessed by the Alarm Distress Baby Scale [9] and is defined by a score of 5 or higher. Persistent (both repetitive and accumulated) sustained social withdrawal has been shown to be a risk factor for altered emotional development in infants [10].

Sustained social withdrawal has typically been associated with severe pathological conditions in infancy such as posttraumatic stress disorder [4], autism spectrum disorders [11], and child depression [12]. Infants demonstrating sustained social withdrawal have a higher risk of developing attachment disorders [13], emotional or behavioral disorders [14], motor and language delays [15], and altered interactive skills [10,16]. Sustained social withdrawal has also been linked with medical conditions such as intrauterine growth retardation, preterm birth [17], early cardiac surgery [18], Prader-Willi syndrome [19], and cleft lip and palate [20], as well as factors in family medical history such as both parents having mental health problems [2]. Sustained social withdrawal has proven to be an important indicator of infant distress regardless of the cause [10].

The Alarm Distress Baby Scale is a well-validated screening tool designed to assess sustained social withdrawal in infants between 2 and 24 months of age in primary care settings such as routine medical checkups or testing [21]. The infant can be assessed during the interaction with the medical professional which avoids putting pressure on the parents (because of their perception that it reflects their caregiving competence) [22].

### Preterm Infants and Mental Health Developmental Risk

Infants born preterm often spend their first days, weeks, or even months in a neonatal intensive care unit where they undergo numerous painful and invasive procedures [23] and can be submitted to various perinatal stresses. During this period, in which both the development and the life of the neonatal infant can be at risk, the infants and their families must adapt to health care unit protocols with respect to visits and care. In addition, the mental health of the parents may be affected increasing the risk of posttraumatic stress and postpartum depression [24].

Several studies have shown that the prevalence of psychopathologies appeared to be significantly higher in infants born preterm than in infants born full term. So far, most studies have focused on infants born very prematurely and have shown high prevalence of autistic spectrum disorders [25], attention-deficit and hyperactivity disorders [26], attachment disorders [13], emotional problems [27], and social withdrawal behavior [28] and growing recognition of behavioral problems [29], lower socioemotional competence [30], and developmental delay [31] relative to prevalence of these in infants born moderately and late preterm. Furthermore, associations between late preterm birth and poor socioeconomic outcomes in adulthood [32] and late preterm birth and poor neurocognitive functioning in late adulthood [33] have been described.

In their first year (corrected for gestational age), infants born preterm have a higher probability of developing sustained social withdrawal (adjusted odds ratio 1.84, 95% CI 1.04-3.26) when compared to that of infants born full term [17] (prevalence was 22.1% and 13.9% for infants born preterm and born full term, respectively). Other studies have found prevalences of sustained social withdrawal that vary from 11.3% to 22.1% for infants born preterm [6,13,17,34].

### Chilean Preterm Infants: Early Detection and Intervention

In Chile, preterm birth rates have been increasing over the last decade. In 2016, 8.3% of live births were preterm (gestational age less than 37 weeks) [35]. Neonatal medical care is provided for these infants (if needed) by a national network of 29 neonatal intensive care units, and follow-up care is provided by interdisciplinary teams in 35 preterm polyclinics. Within the public health system in Chile, all preterm infants are assessed with the Psychomotor Development Assessment Scale [36] between the ages of 0 and 2 years.

Since intervention becomes more challenging as problems in infancy grow more complex or more severe [37], assessing the effectiveness of early sustained social withdrawal detection in preterm infants (using the Alarm Distress Baby Scale) as a precursor to early intervention appears to be an interesting goal.

The Alarm Distress Baby Scale [9] can be used as early as two months of age (corrected age in the case of infants born preterm) and has demonstrated acceptable levels of specificity and sensitivity in several studies (reported in a review study) [10]. In a study with full-term infants, Bonifacino et al [38,39] described a significant difference in sustained social withdrawal in the infants who were followed by Alarm Distress Baby Scale–trained pediatricians compared to sustained social withdrawal in infants who attended routine pediatric follow-up care. Facchini et al [40] investigated the feasibility of a feedback guidance intervention performed by Alarm Distress Baby Scale–trained pediatricians on infants born full term at public well-baby clinics in Italy, and they concluded that these interventions are easily implemented and accepted by patients and their families; however, to the best of our knowledge, there are no reports regarding the effect of an interactive guidance intervention performed by Alarm Distress Baby Scale–trained neonatologists on infants born moderately or late preterm during their first twelve months (corrected age).

The main objective of this study is to compare the effect of an interactive guidance intervention on sustained social withdrawal scores in infants born moderately or late preterm compared to those of infants born moderately or late preterm in routine pediatric care.

## Methods

### Study Design

The study is designed as a randomized controlled trial (NCT03212547), in order to remove bias in treatment allocation and the effect of possible confounding variables [41]. Chilean infants who were born moderately preterm or late preterm (up to N=110) and their parents will be included and randomized in a 1:1 allocation ratio to an intervention group (up to n=55) and a control group (up to n=55). Both groups will receive routine pediatric care at medical checkups at 2, 6, and 12 months of age. All ages used in this study refer to corrected age. To calculate corrected age, the difference in weeks of gestation at the time of birth and full term (40 weeks of gestation) is subtracted from the infant’s chronological age. In addition, the intervention group will receive the interactive guidance intervention, performed by Alarm Distress Baby Scale–trained neonatologists, if the neonatologists detect sustained social withdrawal (a score of 5 or higher in the Alarm Distress Baby Scale) during the routine medical checkups. The interactive guidance intervention will follow a standardized protocol designed by the research team before the start of the recruitment. The control group will not receive the interactive guidance intervention during the first 12 months; however, if any infant in the study receives a score of 5 or higher on the Alarm Distress Baby Scale at the 12-month checkup, they will be offered further assessment and intervention.

### Inclusion Criteria

Only preterm infants born from single or twin pregnancy (monochorionic or dichorionic), born between 32 weeks 0 days and 36 weeks 6 days gestation (as determined by neonatologist), hospitalized within the first 48 hours after birth, and remaining at least 48 hours in the neonatal intensive care unit were eligible to participate in this study. Parents of the infants were required to be Spanish speaking and have stable living arrangements to ensure the effectiveness of the interactive guidance intervention (carried out by Spanish-speaking neonatologists). Parents of infants born preterm were recruited from two neonatal intensive care units—Clinica Alemana de Santiago and Hospital San Jose—by a principal investigator or co-investigator of the study or by the neonatal study coordinator. Clinica Alemana de Santiago is a private health center located in a district of Santiago, Chile with a poverty rate of 0.1% whereas Hospital San Jose is a public health center located in a district of Santiago, Chile with a poverty rate of 8.2% [42,43]. At enrollment, socioeconomic variables were recorded using a socioeconomic survey, and pregnancy and postpartum variables were recorded using a perinatal background questionnaire.

### Exclusion Criteria

Infants were not eligible for participation in this study if their mother had history of or confirmed exposure to cocaine, marijuana, or other illicit mind-altering substances during pregnancy; if the infant had a neurological disease that impairs development that was confirmed at birth; if the infant had major congenital malformations, suspected or confirmed genetic disorders; and if perinatal asphyxia occurred at birth (defined as an Apgar score less than 3 at 1 minute or an Apgar score less than 5 at 5 minutes, or cord pH less than 7.0 at birth).

### Protocol

For this study, in 2017, Bonifacino et al [38] conducted Alarm Distress Baby Scale training in Chile over 4 days which consisted of 30 hours and 12 training modules. The program featured 2 central theoretical modules which covered early interactions and emotional development, emotional deprivation and its consequences, social withdrawal behaviors as an early alarm sign, and the Alarm Distress Baby Scale fundamentals. Subsequently, 10 modules of video training were presented featuring material with infants between 2 and 24 months of age. These infants had attended medical checkups with Alarm Distress Baby Scale–trained and untrained pediatricians. Following the videos, there was an exam to certify the professionals in the use of the Alarm Distress Baby Scale. Fleiss kappa was used to determine if there was agreement between the 11 evaluators regarding diagnosis (normal behavior or sustained social withdrawal). There was a substantial agreement [44] between evaluators (κ=0.794, 95% CI 0.676-0.913; *P*˂.001). Individual kappa values for normal and sustained social withdrawal categories were κ=0.795 and κ=0.794, respectively.

One fundamental element of the training was that the professionals learned not only to detect the sustained social withdrawal behaviors (and to score these behaviors using the Alarm Distress Baby Scale), but also to meaningfully show the parents how—in terms of communication or contact—their infants seek interaction, with the objective of reducing sustained social withdrawal behaviors.

Participants of the study will be recruited during their admission to the neonatal intensive care units by members of the researcher team and enrolled and randomized by a study coordinator to either the intervention or the control group. The infant will be randomized in a 1:1 allocation using SPSS Statistics (version 25.0; IBM Corp), stratified by hospital center (Clinica Alemana de Santiago or Hospital San Jose) in blocks of four. Also, infants will be stratified into two groups, single or twin pregnancy, in order to isolate the intervention effect from that of other covariables (such as mother of twins learning). The Hospital San Jose sample will also be stratified into two groups, whether the infant is included or is not included in the Kangaroo Care program, since only Hospital San Jose currently offers this program that aims to promote mother infant bonding, and which could potentially act as a confounding variable.

**Figure 1:**
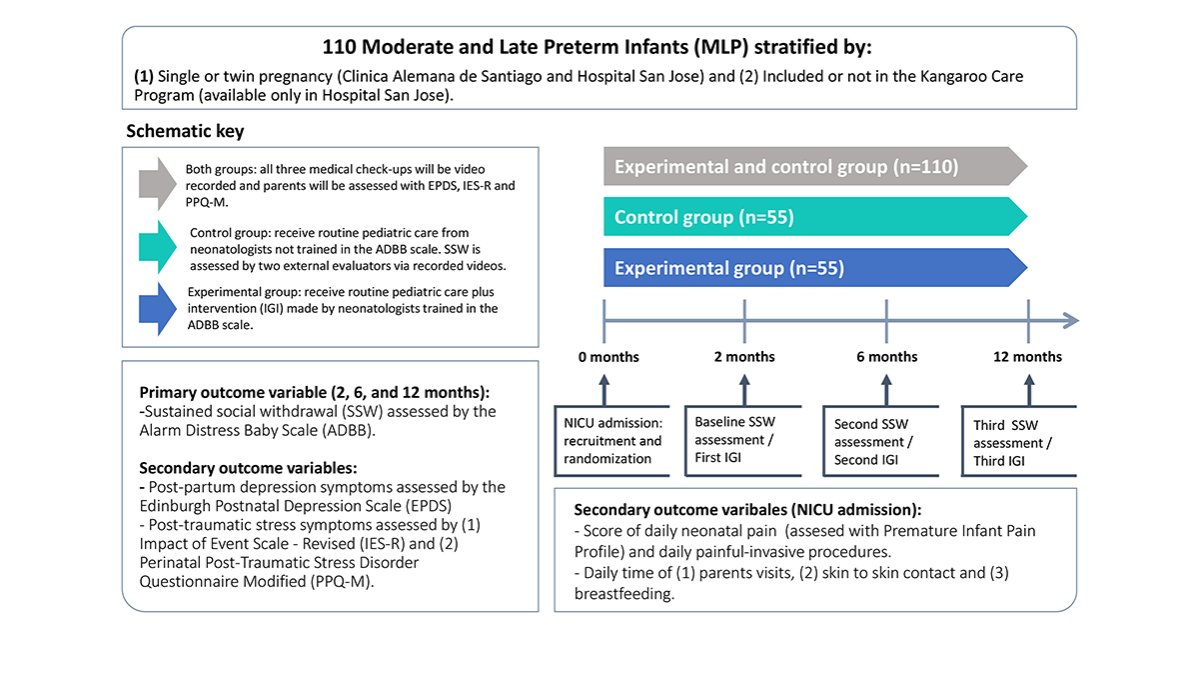
Interactive Guidance Intervention plan for infants born moderately or late preterm with primary and secondary outcome variables recorded during NICU admission and during medical checkups at 2, 6, and 12 months of corrected age. ADBB: Alarm Distress Baby scale; EPDS: Edinburgh Postnatal Depression scale; IES-R: Impact of Event scale–revised; IGI: Interactive Guidance Intervention; NICU: neonatal intensive care; PPQ-M: modified Perinatal Posttraumatic Stress Disorder Questionnaire; SSW: sustained social withdrawal.

As shown in Figure 1, all infants will receive routine pediatric care during the medical checkups at 2, 6, and 12 months of age, but in addition, infants in the intervention group will also receive the interactive guidance intervention if the Alarm Distress Baby Scale–trained neonatologists detect sustained social withdrawal during these medical checkups. All medical checkups will be video-recorded, and the videos will be assessed by external Alarm Distress Baby Scale–trained evaluators, and at every medical checkup, parents will fill out the Edinburgh Postnatal Depression Scale, the modified Perinatal Posttraumatic Stress Disorder Questionnaire, and the Impact of Event Scale–revised.

### Blinding

#### Participants

The families of the infants born moderately or late preterm, randomly assigned to intervention or control group, will remain blind to which group they belong. Families will receive feedback on their infant’s Alarm Distress Baby Scale score by telephone after the final assessment at the 12-month medical checkup and will be offered further assessment and intervention, if needed.

#### Care Providers

Neonatologists who care for participants during the trial will be aware of whether the infant is in the intervention or control group. The Alarm Distress Baby Scale–trained neonatologists will only perform follow-up for the intervention group, and neonatologists who provide routine pediatric care will only perform follow-up for the control group.

#### Investigator

The principal investigator (JBL) will also be an external evaluator. Though, an Alarm Distress Baby Scale–trained psychologist, JBL will not participate in the follow-up of any of the infants and will be blind to the Alarm Distress Baby Scale scores of the Alarm Distress Baby Scale–trained neonatologists. Scores will be entered into the database by an independent agent who is blind to the group category.

#### Outcome Assessment

The Alarm Distress Baby Scale–trained neonatologists will be blind to the Alarm Distress Baby Scale scores of the external evaluators and to those of the other Alarm Distress Baby Scale–trained neonatologists. Once they record an Alarm Distress Baby Scale score, this data will be collected by a study coordinator, who is blind to the group category.

#### Data Collection Assessors

Data collection assessors will collect all study data (all variables) and upload the data into the database. They were not trained in the Alarm Distress Baby Scale, are blind to the group category, and do not have contact with the infants or their families.

### Sample Size

We used the G*Power 3 software (Psychonomic Society Inc) to determine the minimum sample size required for obtaining a significant medium effect size (an effect size of 0.25), given α=.05 and a statistical power of 0.80 (β=.20), using the results presented in Bonifacino et al [37]. In Bonifacino et al’s study [37], the dependent variable was assessed in 4 different stages, with 3 of them involving the total sample. Also, between the second and third assessments, the control group received the intervention. Because of this, we considered the observed differences between the first and second assessments in order to compute our sample size (postintervention difference in prevalence of 57% for the control group and 13% for the intervention group). The resulting sample size estimate was 23 per group; however, in order to ensure a sufficient effect size and taking attrition into consideration, a sample size of 55 infants per group will be used.

### Assessment Instruments

Alarm Distress Baby Scale [9] reliably (Cronbach α=.83) assesses sustained social withdrawal behavior in infants from 2 to 24 months of corrected age during routine physical examination. It consists of 8 items (lack of facial expression, eye contact, general movement, self-stimulation gestures, vocalization, liveliness in response to any stimulation, ability to establish and maintain a relationship, and ability to attract and catch the attention of others) each scored from 0 (normal behavior) to 4 (massively abnormal behavior). A total score of 5 or more indicates sustained social withdrawal behavior. The assessment can be done during routine pediatric checkup by a trained professional, or by assessment of an 8 to 12-minute video of recorded infant behavior during a pediatric checkup.

Edinburgh Postnatal Depression Scale [45] reliably (Cronbach α=.77) assesses the probability of postnatal depression in women. It consists of 10 questions with 4 possible answers for each. Each answer is given a score of 0, 1, 2, or 3 according to the severity of the symptom. The maximum score is 30. A total score of 12 or higher suggests the presence of a postnatal depression disorder. The Edinburgh Postnatal Depression Scale can be administered from 2 months onward, after delivery.

Impact of Event Scale–revised [46] reliably (Cronbach α=.98) assesses symptoms associated with posttraumatic stress disorder. It is composed of 22 items and three subscales: intrusion, avoidance, and hyperactivation. It employs a 5-point Likert scale from 0 (not at all) to 4 (extremely), to assess the intensity of the symptoms. The Impact of Event Scale–revised can be applied from six weeks onward, after the occurrence of a stressful or traumatic event. Scores higher than 24 indicate significant clinical relevance.

Modified Perinatal Posttraumatic Stress Disorder Questionnaire [47] reliably (Cronbach α=.90) assesses posttraumatic stress symptoms in parents including intrusiveness or reexperiencing, avoidance behaviors and hyperarousal, or numbing of responsiveness. It consists of 14 items, which are measured using a 5-point Likert scale from 0 to 4. Parents are instructed to provide responses that reflect their experience during the 4th and 18th month after delivery. The total score can range from 0 to 56. The clinical range for a high-risk parent is set at 19 or higher.

Premature Infant Pain Profile [48] is a reliable (Cohen κ≥0.85, consistently) 7-indicator composite measure developed to assess acute pain in preterm and term neonates . The 7 indicators are gestational age, behavioral state, heart rate, oxygen saturation, brow bulge, eye squeeze, and nasolabial furrow. Each indicator is numerically scored using a 4-point Likert scale from 0 to 4, and total scores can range from 0 to 21. Higher total scores indicate greater pain. Premature Infant Pain Profile scores below 6 means no pain, 6 or higher indicates pain, and 12 or higher indicates moderate to severe pain.

### Intervention

**Figure 2:**
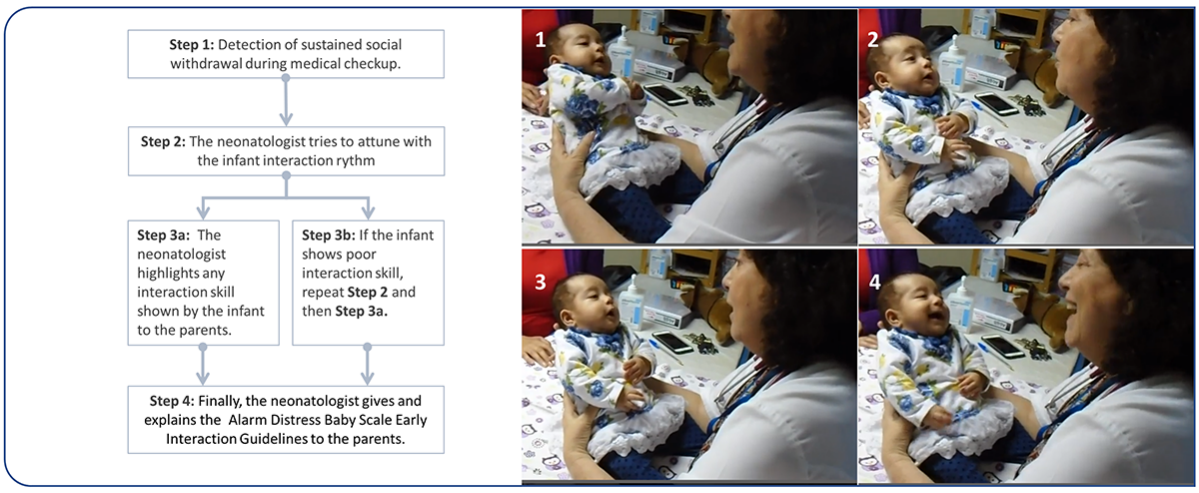
Summary of the steps of the interactive guidance intervention during medical checkup.

As shown in Figure 2, this interactive guidance intervention consists of a brief, nonintrusive, behaviorally focused intervention performed during medical checkup and which follows a simple protocol that is based on the intervention proposed by Bonifacino et al [38].

The main objective of this intervention is to reduce sustained social withdrawal behaviors in moderately and late preterm infants by (1) detecting sustained social withdrawal behaviors during medical checkup, (2) becoming attuned with the interaction rhythm of the infant, (3) meaningfully showing parents how their infant seeks to interact by pointing out specific interactive behaviors (every interaction skill displayed by the infant), and ( 4) inviting parents to engage in the interaction.

Infants born preterm have a higher probability of developing sustained social withdrawal, and withdrawn infants show poor performance during interaction [17,49]; therefore, by facilitating parental understanding of the development of their infant, by involving them in the observation of and interaction with their infant [50], and by reinforcing every seeking interactive behavior displayed by the infant during medical checkups, we can reduce sustained social withdrawal in these infants.

In the intervention group, this interactive guidance intervention will be performed if the trained neonatologist detects sustained social withdrawal during the 2, 6, and 12-month medical checkups. The interactive guidance intervention will be performed during the 20 to 30-minute medical checkup and will not require any extra time. The interactive guidance intervention will be supplemented with a written guide for parents called Early Interaction Guidelines. The objective of this written guideline is to enhance the effect of interactive guidance intervention.

In the control group, infants will receive routine pediatric care. At the 2, 6, and 12-month medical checkups (also lasting 20 to 30 minutes), parents will be given a Development Stimulation Guide which has been adapted from Ministry of Health of Chile Guidelines for the Stimulation of Development [51,52].

If any infant in the study (in either the intervention or the control group) shows sustained social withdrawal (a score or 5 or higher on the Alarm Distress Baby Scale) at the 12-month medical checkup, they will be offered additional interventions by the researcher team.

### Outcomes

#### Primary Outcome Measure

The primary outcome is sustained social withdrawal assessed using the Alarm Distress Baby Scale. The Alarm Distress Baby Scale categorizes the level of sustained social withdrawal according to the sum of the score—scores from 0 to 4 indicate no sustained social withdrawal, scores from 5 to 9 indicate moderate sustained social withdrawal, and scores equal to or higher than 10 indicate severe sustained social withdrawal. Infants born moderately or late preterm will be assessed with the Alarm Distress Baby Scale during medical checkups at 2, 6, and 12 months of age.

#### Secondary Outcome Measures

The secondary outcomes measured during neonatal intensive care unit admission are neonatal pain and daily duration of parental visits, skin-to-skin contact, and breastfeeding; and the secondary outcomes measured after medical discharge are postpartum depression symptoms and posttraumatic stress symptoms which will be assessed at the 2, 6, and 12-month medical checkups. A socioeconomic survey, a substance-use survey, and a perinatal background questionnaire will be used to obtain additional information from the parents of the infants.

### Statistical Analysis

Two-tailed paired *t* tests will be used to compare covariates and dependent variables from the infant groups. A one-way analysis of covariance will be used to determine the effects of covariates (sociodemographic and neonatal intensive care unit hospitalization variables, postpartum depression, and posttraumatic stress) on the comparisons between groups (control and intervention) and between times (2, 6, and 12 months of corrected age medical checkups) regarding the dependent variable (sustained social withdrawal). Other statistical techniques such as matching, odds ratios, and logistic regression will also be used.

### Ethics

The study was approved by the *Comité Ético Científico de la Facultad de Medicina* (Scientific and Ethics Committee of the Faculty of Medicine), Universidad del Desarrollo (approval record 2017-05) on April 25, 2017. The clinical trial was registered at the United States National Institutes of Health (clinicaltrials.gov; NCT03212547) and approved on July 7, 2017. Informed oral and written consent were obtained from the accompanying parent of each infant included in the study.

## Results

The clinical trial is ongoing. It was funded in December 2016, approved by institutional review board on April 25, 2017. Data collection started on September 19, 2017. As of May of 2020, enrollment has been completed (N=110 infants born moderately or late preterm). We aim to publish the results by mid-2021. The data sets from this study will be available by request from the corresponding author, once results have been published.

## Discussion

### Summary

This project is a logical continuation of the work in Bonifacino et al [38] and aims to contribute to the early detection of alarm signs in emotional development and early intervention to help infants born moderately or late preterm. Neonatologists have a central role to play in this regard, and based on the findings of both Bonifacino et al [38] and Facchini et al [40], we hypothesize that implementing interactive guidance intervention will reduce sustained social withdrawal in infants born moderately or late preterm compared to those who receive routine care. Also, we hypothesize that this interactive guidance intervention will be easily implemented and accepted by patients and their families.

This is the first randomized controlled trial that will be performed with infants born moderately or late preterm at follow-up pediatric checkups. The implementation of an interactive guidance intervention will allow Alarm Distress Baby Scale–trained professionals who use this interactive guidance intervention as a model to intervene for infants at risk.

### Study Limitations

One of the limitations of this study is the possibility that the parents of infants included in the intervention group will share information with parents of infants included in the control group. For this reason, twins are randomized together (both either in the control or in the intervention group). Additionally, all parents included in the study will be asked to refrain from sharing the written guides used in the study with others until the study is finished.

Another limitation is the pain protocol established at each institution. Clinica Alemana de Santiago has had a standardized pain protocol since 2012 that includes administering the Premature Infant Pain Profile every 3 hours while, in Hospital San Jose, the standardized pain protocol does not include the Premature Infant Pain Profile. Before the start of study recruitment, training was performed by nurses of Clinica Alemana de Santiago in order to teach the midwives in Hospital San Jose how to administer the Premature Infant Pain Profile. At Hospital San Jose, this is only done for the infants included in the study.

### Ethical Considerations

The interactive guidance intervention does not involve any risk to the participating infants and parents. The neonatologists will follow routine medical care protocols at medical checkups and will only perform the interactive guidance intervention (verbally) if they detect sustained social withdrawal. Currently, the neonatologists on the investigation team are the only follow-up neonatologists trained in the Alarm Distress Baby Scale in Chile, so these infants will receive an evaluation and an intervention that is otherwise not currently available in either the public or in the private health system. Finally, if any infant scores 5 or higher on the Alarm Distress Baby Scale at the 12-month medical checkup (final assessment), they will be offered further assessment and intervention.
